# In Silico Analysis of the ROP29 Protein as a Vaccine Candidate Against *Toxoplasma gondii*

**DOI:** 10.1155/2024/1918202

**Published:** 2024-07-26

**Authors:** Amir Karimipour-Saryazdi, Fatemeh Ghaffarifar, Abdolhossein Dalimi, Masoud Foroutan, John Horton, Javid Sadraei

**Affiliations:** ^1^ Department of Parasitology Faculty of Medical Sciences Tarbiat Modares University, Tehran, Iran; ^2^ Department of Basic Medical Sciences Faculty of Medicine Abadan University of Medical Sciences, Abadan, Iran; ^3^ Tropical Projects, Hitchin, UK

**Keywords:** bioinformatic analysis, ROP29, *Toxoplasma gondii*, vaccine

## Abstract

The progression of *Toxoplasma gondii* (*T. gondii*) invasion is aided by rhoptry proteins (ROPs), which are also crucial for the parasite's survival in host cells. In this study, in silico analysis was performed to examine the various aspects of the ROP29 protein, such as physicochemical properties, potential T- and B-cell epitopes, and other significant features. The research revealed that there were 55 possible sites for posttranslational modification in the ROP29 protein. The secondary structure of the ROP29 protein consists of a random coil, an alpha-helix, and an extended strand, which account for 49.69%, 36.81%, and 13.50%, respectively. Moreover, a number of putative T- and B-cell epitopes for ROP29 were found. The Ramachandran plot showed that 88.91% (crude model) and 97.54% (refine model) of the amino acid residues were located in the favored regions. Also, the testing of this protein's antigenicity and allergenicity showed that it was nonallergenic and immunogenic. Our results suggested that employing in silico tools to apply structural and functional predictions to the ROP29 protein can lower the likelihood that laboratory investigations will fail. This research served as a crucial foundation for further research. More research is required in the future in suitable animal model employing ROP29 alone or in combination with other antigens.

## 1. Introduction

Toxoplasmosis is a dangerous zoonotic illness that results from the parasite *Toxoplasma gondii* (*T. gondii*) and infects one-third of the global population [1–3]. Severe and deadly organ damage can be caused by *T. gondii* [4]. Toxoplasmosis is caused by tachyzoites, which are actively dividing and invading in tissues of the host [5, 6]. Humans can become contaminated via eating oocysts shed by cats and through congenital infection as well as eating contaminated meat with tissue cysts [7]. Bradyzoites and tachyzoites are two of these pathogenic types that are connected to the disease's chronic and acute stages, respectively [8]. *T. gondii* is divided into three primary clonal lineages, Genotypes I, II, and III, which vary from one another in terms of virulence, pathogenicity, and epidemiological patterns [9, 10]. The largest number of human infections in Europe and North America are caused by Type II strains. Interestingly, farm animals from these regions tend to have this genotype as well [11]. Although *T. gondii* frequently causes subclinical infection, it can sometimes result in serious consequences in immunocompromised people and neonates with congenital infectious disease, including blindness, mental retardation, hydrocephalus, and encephalitis [12, 13]. Furthermore, livestock is the primary source of infection for humans, and toxoplasmosis infection may result in significant economic losses because of abortions, neonatal mortality, and stillbirths [14].

There are few interventions available to track the tachyzoite stage despite our growing understanding of *T. gondii* [15]. The drugs used to treat toxoplasmosis only restrict the formation of *T. gondii* tachyzoites at the beginning of infection; they are unable to remove encysted parasites from infected hosts. These drugs have side effects. Additionally, there are no cures to get rid of parasite from the host [16]. Additionally, immunization against this parasite is another way to keep tabs on toxoplasmosis [17]. Due to the significance of both economic losses in the livestock industry and global public health, the creation of effective vaccines against this parasite is a key objective [18]. Recent years have seen a large number of antigens described as vaccine candidates; nevertheless, there are few that exhibit high resistance and long-term protection. As a result, selecting appropriate antigens is a critical first step in creating reliable and effective vaccinations [19, 20]. The most effective vaccination candidates for *Toxoplasma* are rhoptry proteins (ROPs), microneme proteins (MIC), surface antigens (SAGs), and dense granules (GRA) according to the results of numerous research [21–24] The gene family of ROPs encodes a number of significant and essential proteins that contribute to *Toxoplasma* pathogenesis [25, 26]. They play an important role in many stages of parasite invasion and are essential for survival in the host cells [27–29]. Nearly 1%–30% of all parasite cell mass is made up of rhoptries [30]. Several rhoptry antigens, including ROP8, ROP21, ROP29, and ROP16, are exceedingly pathogenic and act as virulence factors. This protein, like ROP18 and ROP16 proteins, has a high antigenicity, which strongly stimulates the host's cellular immune system [31–34].

Bioinformatic approaches are useful for the selection of immunodominant epitopes since those have already been utilized to determine the potential of T- and B-cell epitopes as vaccination candidates [35]. These tools have been utilized on a regular basis to measure gene and protein expression as well as structural prediction, immunogenic, and other protein features. Our understanding of proteins can be improved by studying and analyzing their chemical, physical, and immunogenic features, which can also assist researchers in choosing the best epitopes for vaccine research [36, 37]. When compared to conventional methods, bioinformatics has a number of benefits, such as high accuracy and precision relative time and cost-effectiveness [20, 36]. So, for diagnostic purposes and vaccine development, the detection of protein epitope features using bioinformatic approaches will be helpful [38]. Because of this, the present study's objective was to use a variety of bioinformatics web servers to examine physicochemical characteristics, the structure, transmembrane domains, subcellular localization and phosphorylation sites, and immunogenicity of the ROP29 protein of *T. gondii* parasite.

## 2. Materials and Methods

### 2.1. Sequence Accessibility

Prior to bioinformatic analysis, the entire amino acid sequence of ROP29 was recovered in FASTA format from database in the National Center for Biotechnology Information and available online (https://www.ncbi.nlm.nih.gov/protein/).

### 2.2. Evaluation of Physicochemical Characteristics

The ROP29 protein's various physicochemical parameters, including its amino acid composition, theoretical isoelectric point (pI), total number of positively and negatively charged residues, aliphatic index, molecular weight (MW), instability index, estimated half-lives in vivo and in vitro, grand average of hydropathicity (GRAVY), and extinction coefficients, were analyzed using the ExPASy ProtParam web server at https://web.expasy.org/protparam [39].

### 2.3. ROP29 Protein Transmembrane Domain Prediction and Subcellular Location

PSORT II web service (http://psort.hgc.jp/form2.html) also anticipated the ROP29 protein subcellular site. To assess the transmembrane domains of ROP29, the TMHMM online server v.2.0 (https://services.healthtech.dtu.dk/services/TMHMM-2.0/) was used [40].

### 2.4. ROP29 Protein Phosphorylation and Acylation Locations

NetAcet-1.0 (https://services.healthtech.dtu.dk/services/NetAcet-1.0) and NetPhos 3.1 (https://services.healthtech.dtu.dk/services/NetPhos-3.1/) online tools were used to evaluate the acetylation and phosphorylation locations of ROP29 protein, respectively [40].

### 2.5. Prediction of Secondary Structure

Garnier–Osguthorpe–Robson 4 (GOR4) online server [41] was used to predict the ROP29 protein secondary structure. Furthermore, the probability of *β*-sheet, *α*-helix, or random coil was calculated for each amino acid position using the GOR4 online server. Moreover, the DiNNNA web tool (unified program for prediction of disulfide bond partner and cysteine state) was employed to forecast disulfide bonds (http://clavius.bc.edu/clotelab/DiANNA/) [42, 43].

### 2.6. Building a Three-Dimensional (3D) Model

A crucial step in the design of vaccines is the generation of 3D model structures. 3D models of the ROP29 sequence were made using the web-based homology modeling tool SWISS-MODEL (https://swissmodel.expasy.org/), which is believed to foresee protein 3D constructs with many amino acids [30, 44].

### 2.7. Validation and Fine-Tuning of the 3D-Modeled Structure

The best model (generated by SWISS-MODEL) was chosen and modified with GalaxyRefine at http://galaxy.seoklab.org/cgi-bin/submit.cgi?type=REFINE to enhance the accuracy of the template-based protein system forecast. Initially, the GalaxyRefine server uses molecular dynamic simulation to rebuild side chains, which it then repacks and uses to relax the overall structure [45]. The Ramachandran plot was used to verify the protein's 3D shape using the SWISS-MODEL tool at https://swissmodel.expasy.org/assess [46]. An economically favored portion of a backbone dihedral angle is shown versus amino acid residues in a protein structure using a Ramachandran plot (https://swissmodel.expasy.org/assess/help). Additionally, ProSA-web was utilized to evaluate the model's general quality (https://prosa.services.came.sbg.ac.at/prosa.php) [47].

### 2.8. ROP29 Protein Discontinuous (Conformational) and Continuous (Linear) B Lymphocyte Epitopes

To find the continuous B-cell epitopes, the BCPREDS was used. Multiple prediction program was utilized to examine the B-cell linear epitopes. Various servers were used to anticipate the ROP29 protein's B-cell epitopes. First, attempt BcePred (B lymphocyte cell epitope prediction) at http://crdd.osdd.net/raghava/bcepred/bcepred submission.html. Physicochemical properties were utilized to predict B-cell linear epitopes. Users of this website (http://crdd.osdd.net/raghava/bcepred/) may foresee B-cell epitopes using any of the physical and chemical factors (mobility, hydrophilicity, accessibility, flexibility, turns, polarity, and exposed surface), with the maximum precision of 58.70% at a threshold of 2.38 [48, 49]. The BcePred server was used with the default parameters for forecast. We also employed the ABCpred (artificial neural network [ANN]-based B-cell epitope prediction) website [45, 47]. The ABCpred web service's goal is to identify B-cell epitopes in an antigen sequence using an ANN (http://crdd.osdd.net/raghava/abcpred/). The anticipated epitopes of B-cell had an amino acid length of 20, and the threshold was 0.75%. Furthermore, the immune epitope database (IEDB) was used to predict antigenicity [50], flexibility [51], linear epitope prediction [52], surface accessibility [53], hydrophilicity [54], and beta-turn [55]. The DiscoTope 2.0 website (http://www.cbs.dtu.dk/services/DiscoTope/) was then utilized to predict conformational epitopes of B-cell from the 3D structure of protein [56]. This web service, in short, employs a surface accessibility assessment (calculated in terms of contact numbers) and unique score of epitope propensity amino acid. For epitope forecasting, the default value (level: 3.7) was employed. The specificity is 0.75 and the sensitivity is 0.47 at this point (http://www.cbs.dtu.dk/services/DiscoTope/instructions.php).

### 2.9. Detection of MHC-I and MHC-II Epitopes

Based on the alleles of the BALB/c mice strain, the IEDB web server was utilized to evaluate the affinity of ROP29 protein that interact with the MHC Class II (http://tools.iedb.org/mhcii/) and Class I (http://tools.iedb.org/mhci/) molecules. Mouse MHC-I molecules were selected from the alleles, H2-Kk, H2-Db, H2-Dd, H2-Kb, H2-Ld, and H2-Kd. Furthermore, the forecasting was carried out with 10-amino-acid-long peptides utilizing the IEDB-recommended approach. In addition, three mouse MHC-II alleles (H2-IAd, H2-IEd, and H2-IAb) were selected. The IEDB-recommended prediction method was used, and 15 amino acids, sorted by percentile rank, were the predicted length of the peptide.

### 2.10. Forecasting of Cytotoxic T-Cell (CTL) Epitopes

Antigen presentation on the surface of MHC-I is the preliminary stage in stimulating the immune system. As a result, one of the most important aspects of vaccine design is the selection of CTL epitopes. We accomplished this by using the openly accessible web service CTLPred [57], available online at http://www.imtech.res.in/raghava/ctlpred/index.html. A consensus procedure was employed to make the forecast [58]. By default, the support vector machine's (SVM and ANN) cutoff scores were set at 0.36 and 0.51, respectively. The distinction between epitopes and nonepitopes is made using the cutoff value. Reliability for the consensus prediction method was 77.6% (http://crdd.osdd.net/raghava/ctlpred/about.html).

### 2.11. Assessment of Solubility, Allergenicity, and Antigenicity

ANTIGENpro is a database of antigens (http://scratch.proteomics.ics.uci.edu/) [59]. Also, to forecast antigenicity of the ROP29 protein, the VaxiJen v.2.0 (http://www.ddg-pharmfac.net/vaxijen/VaxiJen/VaxiJen.html) [60] web server was used. ANTIGENpro is an estimator of protein antigenicity that is sequence-based, pathogen-independent, and alignment-free (https://scratch.proteomics.ics.uci.edu/explanation.html#ANTIGENpro). A novel approach to antigen detection, VaxiJen v.2.0, is predicated on the autocross covariance (ACC) modification of protein sequences into uniform vectors with significant amino acid properties. Depending on the specific organisms, the web service's efficiency ranges from 70% to 89%.

The SOLpro server (http://scratch.proteomics.ics.uci.edu/) was employed to forecast the solubility of proteins after overexpression [61]. Utilizing a two-step SVM architecture built around several primary sequence representations, SOLpro predicts whether an overexpressed protein in *Escherichia coli* bacteria will be soluble (http://scratch.proteomics.ics.uci.edu/explanation.html#SOLpro). Finally, the AlgPred service (http://www.imtech.res.in/raghava/algpred/) predicted the allergenicity of ROP29 protein [49]. The server can predict allergenicity using six distinct ways. With 85% reliability, at a threshold of 0.4, the hybrid technique (ARPs BLAST + MAST + SVMc + IgE epitope) was utilized.

## 3. Results

### 3.1. ROP29 Protein Basic Analysis

The NCBI's accession number for the ROP29 protein's amino acid sequence in FASTA format is EPT30384.1. The ROP29 protein had a theoretical pI of 6.92, had 489 residues of amino acids, and had a MW of 53077.52 Da. The ROP29 protein had 47 positively (Arg + Lys) and 48 negatively (Asp + Glu) charged residues, respectively. ROP29 included 7500 atoms in total, and its extinction coefficient in water at 280 nm was 53,775 M^−1^ cm^−1^. Mammalian reticulocytes in vitro were estimated to have a half-life of 30 h, yeast in vivo of more than 20 h, and *E. coli* in vivo of more than 10 h. The protein's instability index (II) was estimated to be 39.66, indicating that it is stable. ROP29 protein's aliphatic index and GRAVY were 91.41 and −0.086 respectively.

### 3.2. ROP29 PTM Site Detection

**Figure 1:**
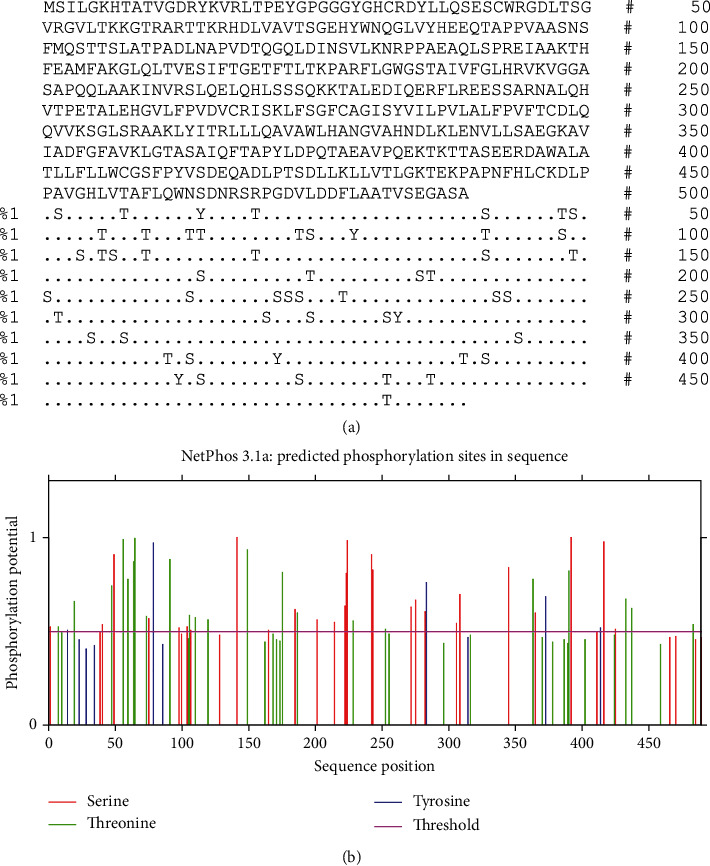
Bioinformatic analysis of phosphorylation and sites of ROP29. (a) The location is indicated by a dot (“.”) if the residue is expected not to be phosphorylated, either because the score is below the threshold or because Ser, Thr, or Tyr are not present (https://services.healthtech.dtu.dk/services/NetPhos-3.1/). Residues with a prediction score over the threshold are denoted by the letters “S,” “T,” or “Y,” respectively; (b) phosphorylation sites of ROP29 protein.

In this study, we analyzed the ROP29 protein acetylation and phosphorylation locations using the web tools NetAcet-1.0 and NetPhos 3.1, respectively. The results revealed 1 acetylation (Table 1) and 54 phosphorylation sites (Thr: 22, Ser: 27, and Tyr: 5) in the ROP29 protein, revealing that the ROP29 sequence has 55 possible PTM locations (Figure 1).

### 3.3. ROP29 Transmembrane Domain Identification and Subcellular Localization

**Figure 2:**
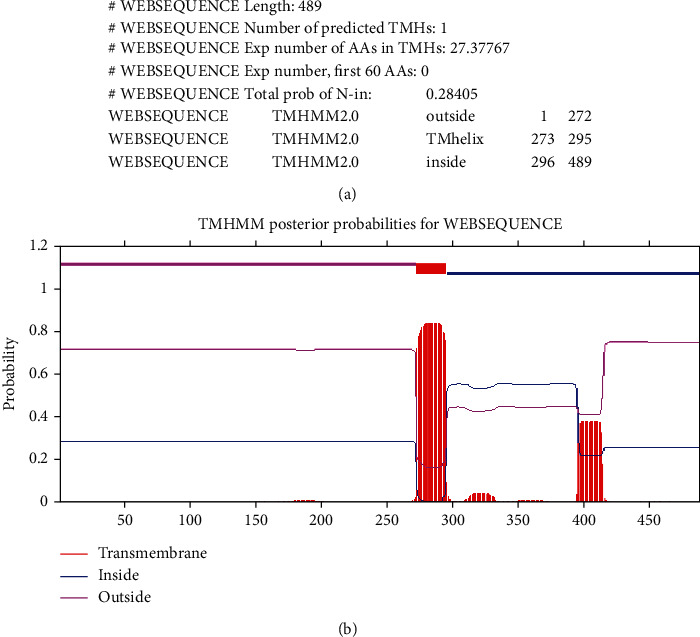
Analysis of transmembrane domain of ROP29 (available at https://services.healthtech.dtu.dk/services/TMHMM-2.0/). (a) Predicted number of TMHs. The estimated number of transmembrane helices and the number of AAs found in TMHs show that many amino acids are anticipated to be in transmembrane helices. A transmembrane protein is very certainly present if this number is higher than 18 (or contains a signal peptide). Exp number, first 60 AAs: the first 60 amino acids of a protein make up the anticipated number of transmembrane helices. You should be informed that a forecasted transmembrane helix in the N-term could be a signal peptide if this number is greater than a few. Total N-in probability: the overall likelihood that the N-term is on the membrane's cytoplasmic side. N-term signal sequence that might be used: a message that appears when “Exp number, first 60 AAs” is more than 10. (b) Examining ROP29's transmembrane domain.

The TMHMM service v.2.0 outcomes revealed that the ROP29 sequence has one transmembrane domain (Figure 2). Furthermore, PSORT II predicted ROP29 subcellular location including 4.3% Golgi, 4.3% vesicles of secretory system, 4.3% peroxisomal, 8.7% endoplasmic reticulum, 26.1% mitochondrial, 17.4% nuclear, and 34.8% cytoplasmic.

### 3.4. Secondary Structure Prediction and Analysis

**Figure 3:**
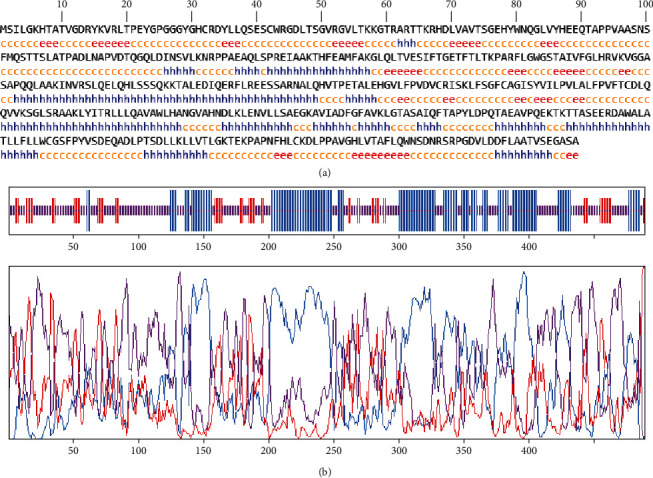
Predicted secondary structure by GOR4 online service (https://npsa.lyon.inserm.fr/cgi-bin/npsa_automat.pl?page=/NPSA/npsa_gor4.html). (a) h = helix, e = extended strand, t = turn, and c = coil. (b) Graphical results of the prediction of secondary structure of ROP29 protein by GOR4.

The GOR4 online site was utilized to examine the ROP29 protein's secondary structure. GOR4 predicts the fundamental components of proteins, including the extended chain, random coil, and alpha-helix. The data from the GOR4 website showed that the alpha-helix, random coil, and extended chain proportions in the ROP29 sequence were 36.81% (180/489), 49.69% (243/489), and 13.50% (66/489), respectively (Figure 3). We used DiANNA application system to estimate disulfide bonds in this investigation. DiANNA (http://clavius.bc.edu/clotelab/DiANNA/) is a comprehensive program for predicting cysteine and disulfide connections. The findings showed that there are seven cysteines in our sequence. At positions 32–42, 268–446, and 297–408, the cysteines are predicted to form the disulfide bond.  Table 2 has more information.

### 3.5. 3D Model Forecast and Assessment

**Figure 4:**
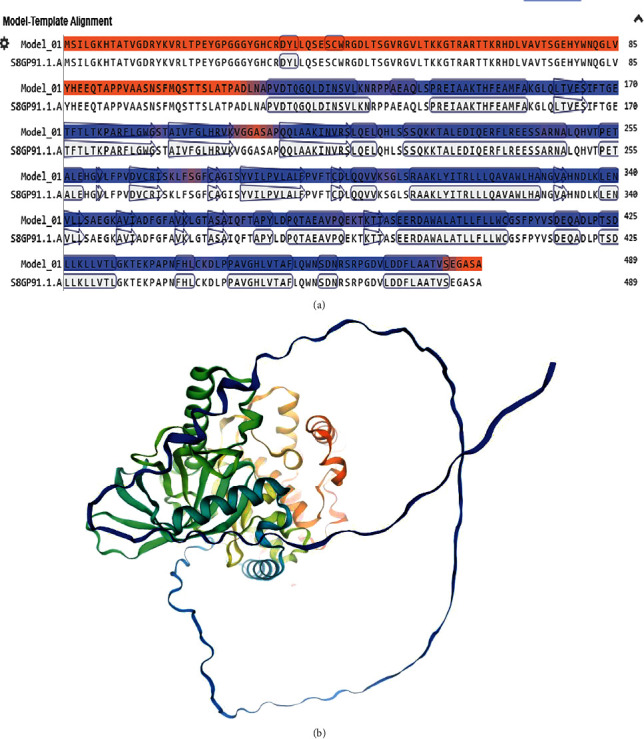
Results from the web server SWISS-MODEL (https://swissmodel.expasy.org/). (a) Model-template alignment. (b) The ROP29 protein's 3D model.

The web server SWISS-MODEL was utilized to create 3D models of the ROP29 protein. Following prediction, three 3D models of the ROP29 sequence were created, and the model with the highest identity was chosen. Among the SWISS-MODEL models, the selected model had 100% sequence identity with coverage of 1.00 from 1 to 489 amino acid residues.  Figure 4 depicts the SWISS-MODEL results in terms of model template alignment and ROP29 protein 3D model.

### 3.6. 3D Structure Improvement and Verification

**Figure 5:**
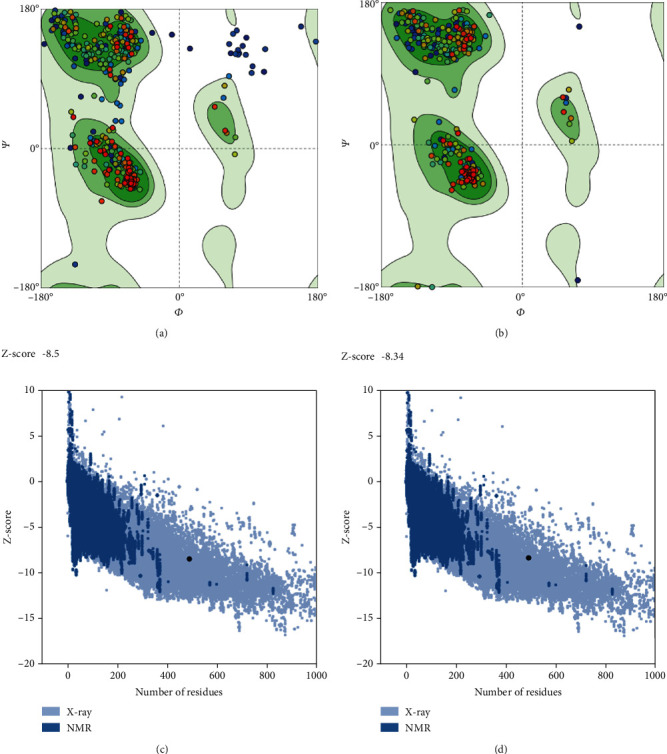
Validation of the three-dimensional model of the ROP29 protein using structure assessment tool and ProSA-web server for (a, c) crude and (b, d) refined models.

The acquired tertiary structures were refined using the GalaxyRefine program. Based on findings, the quality of the 3D structures was enhanced after modification. Protein verification using structure assessment tool prior to refinement revealed that in the initial model, 88.91% of residues were in favored locations. After refinement by GalaxyRefine, according to Ramachandran plot, 97.54% of the total residues were in the favored regions (Figure 5).

### 3.7. ROP29 Protein Discontinuous and Continuous B Lymphocyte Epitopes

**Figure 6:**
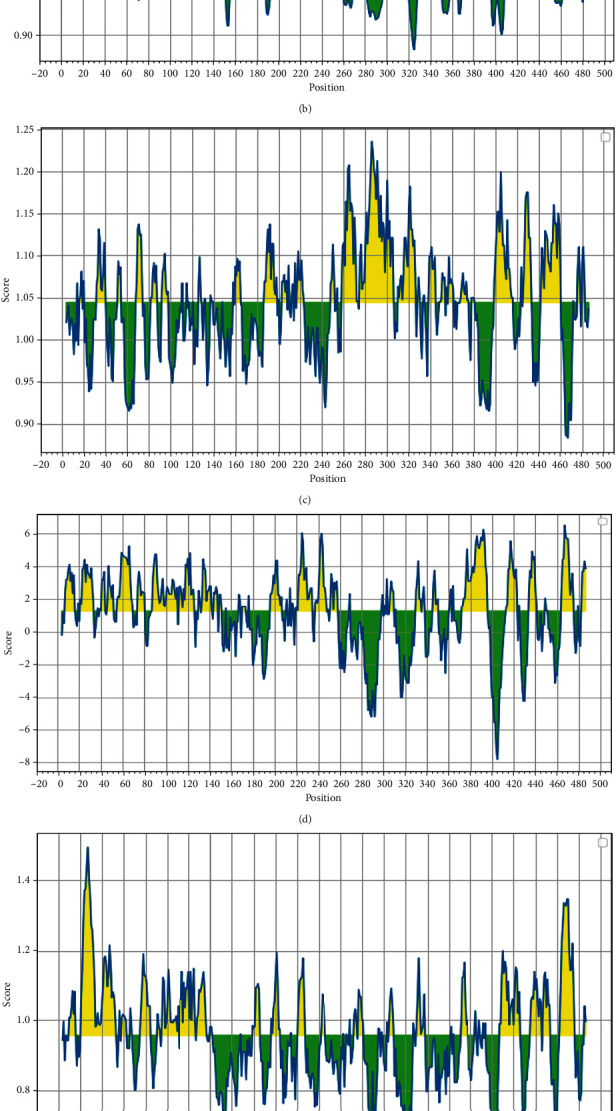
Propensity scale plots of ROP29 protein. (a) Surface accessibility, (b) flexibility, (c) antigenicity, (d) hydrophilicity, (e) beta-turn, and (f) BepiPred linear epitope prediction. The average score or threshold is indicated by the horizontal red line. The favorable regions linked to the features of interest are indicated by yellow colors (above the threshold). The regions associated with the attributes of interest that are unfavorable are shown by green color (below the threshold).

ROP29 protein linear epitopes were predicted using different web servers, including BcePred, BCPREDS, ABCpred, and IEDB. The BcePred was used to categorize B lymphocyte continuous epitopes based on mobility, accessibility, turns, antigenic propensity, exposed layer, polarity, and hydrophilicity.  Table 3 shows the outputs of the BcePred server. Such potential epitopes are crucial for the antigenic characteristics of ROP29. Furthermore, Table 4 displays the outputs of the ABCpred server (length of B-cell epitopes = 20 mer; all peptides in Table 5 are greater than the set threshold level). The greater the peptide's score, the more likely it is to be an epitope. Also, BCPREDS-derived linear B-cell epitopes from full-length ROP29 protein are inserted in Table 5. Using the IEDB online resource, the average beta-turn, antigenicity, BepiPred linear epitope, hydrophilicity, surface accessibility, and flexibility scores for the ROP29 protein were 0.957, 1.044, 0.525, 1.261, 1.000, and 0.996, respectively (Figure 6). In addition, in the 3D model, the DiscoTope 2.0 website was utilized to discover 13 conformational B-cell epitopes.

### 3.8. Forecasting of MHC-I and MHC-II Epitopes

The ROP29 protein's MHC-II and MHC-I binding epitopes for mouse alleles were predicted using the IEDB web database. Bioinformatic investigation revealed that the ROP29 T-cell epitopes can securely attach to MHC-I and MHC-II proteins. Epitopes for ROP29 protein were chosen based on their lowest percentile ranks, showing a stronger affinity for the receptor molecule. Tables 6 and 7 show the minimal percentile ranks of each MHC allele for ROP29.

### 3.9. Forecasting of CTL Epitopes

The CTLPred server was used to forecast CTL epitopes, and CTLPred chose 10 highly ranked and appropriate epitopes (Table 8).

### 3.10. Assessment of Solubility, Allergenicity, and Antigenicity

The ROP29 protein's solubility after overexpression in *E. coli* was estimated to be 0.5919 by the SOLpro website. The nonallergic nature of the ROP29 protein was verified using the AlgPred service. The anticipated antigenicity values of ROP29 protein were 0.7509 using ANTIGENpro and 0.5304 using VaxiJen v.2.0. Both predictions indicate that ROP29 protein is immunogenic (threshold 0.5 was used in VaxiJen).

## 4. Discussion

Toxoplasmosis diseases are a substantial public health concern and have a significant economic impact worldwide [62, 63]. As a result, developing an effective and safe particular vaccination is critical for toxoplasmosis protection [64]. Thus, the first step in developing a viable vaccine is identifying possibly immunoprotective parasite antigens [20]. Finding the functions and composition of new vaccines depends heavily on the investigation of epitopes and structures. Protein bioinformatic approaches have evolved into a crucial tool in vaccine research. These procedures can minimize blindness, are cost-effective, and can significantly cut experimental expenditures [35, 36]. Given the role of ROP proteins in virulence and immunogenicity, these antigens may be suitable vaccine targets against toxoplasmosis [24].

The different characteristics of the ROP29 protein were investigated in this research. We explored several physicochemical features of the ROP29 based on the ExPASy ProtParam server. ROP29 protein's amino acid sequence had 489 residues, and its MW was 53077.52 Da, demonstrating a favorable antigenic nature (weak immunogens are those with a MW of 5–10 kDa) [65]. The protein's durability was assessed using the instability index. As a result of the findings, the ROP29 protein was determined to have an instability index of 39.66, which is considered as stable protein. The GRAVY and aliphatic index of the ROP29 sequence were measured in this study and were found to be −0.086 and 91.41, respectively. In summary, a target protein that has a high aliphatic index is more stable across a larger temperature range. ROP29 was therefore discovered to be thermostable. Additionally, a hydrophilic protein that has a better ability to attach itself to the nearby water molecules is indicated by the negative value of GRAVY.

PTMs are widely established to be essential for cellular regulatory mechanisms [66]. So as to identify the acetylation and phosphorylation locations of the ROP29 protein, we employed two online databases, NetAcet-1.0 and NetPhos 3.1, respectively (https://services.healthtech.dtu.dk/services/NetAcet-1.0/). The findings indicated that the ROP29 had 55 possible PTM sites (one acetylation and 54 phosphorylation sites), suggesting that specific protein activities may be regulated by these sites and that protein activity may be affected. We discovered that although this protein has only one transmembrane domain, antigen-presenting cells can interact with it to prime T- and B-cells and trigger rapid immunological reactions. It should be kept in mind that analyzing the secondary structure of the protein and incorporating unique constraints, such as alpha-helices or beta-turns, are essential steps in predicting the tertiary structure. According to the GOR4 results, the ROP29 protein is made up of 13.50% extended strand, 49.69% random coil, and 36.81% alpha-helix. It is clear that beta-turn and alpha-helix, which are found in the inside of the ROP29 protein and have a high hydrogen bond energy, can maintain the structure of protein, resulting in a solid bond with antibodies [67]. The ultimate goal of protein prediction structures is tertiary structure prediction. Understanding protein structures and the relationships between their functions and structures is critical [20, 30, 68]. The SWISS-MODEL tool was used in this investigation to model the ROP29 protein's tertiary structure. An interactive web-based application called SWISS-MODEL is used to simulate protein structures (https://swissmodel.expasy.org/docs/help). The Ramachandran plot aids in evaluating the validity of evaluating the biological function and using experimental configurations of proteins [69]. Structural validation is another important factor in protein structural forecasting. Using the structure assessment tool, a Ramachandran plot was visualized to confirm the 3D model that had been created. In structural biology, identifying errors in theoretical and experimental models of protein structures is crucial. Due to this, we used SWISS-MODEL to pick the best model, and the GalaxyRefine web server was used to enhance the modeled structure. Finally, the aforementioned method was used to reevaluate the primary and improved models. After refinement, the Ramachandran plot analysis revealed a high-caliber 3D model. The Ramachandran plot's results show that in the first model, 88.91% of residues were in favored locations. However, the GalaxyRefine web server increased the percentages to 97.54%. Due to the high proportion of residues in preferred regions and a low proportion in the outer region, it was therefore assumed that the ROP29 protein constituted a good model.

During the course of infection with *Toxoplasma* parasite, a potent humoral and cell-mediated immunity is developed [70, 71]. To prevent and limit the parasites' ability to bind to the proper host cell receptors, the development of specific-IgG antibodies is necessary. Additionally, it can make it simple for immune cells like macrophages (MQs) to get rid of *Toxoplasma* parasite, which prevents infection from resurfacing [71]. With the help of different T-lymphocytes (such as CD4^+^ and CD8^+^ T-cells) and cytokines such as interferon-*γ* (IFN-*γ*), interleukin-12 (IL-12), interleukin-2 (IL-2), and tumor necrosis factor a (TNF-a), T-cells are in charge of defensive immunity. The regulation of immune responses is greatly aided by other cytokines, such as IL-10, IL-4, and IL-5 [72, 73]. B-cell antibody generation is crucial for the long-term defense against toxoplasmosis, even though CD8^+^ T-cells that produce IFN-*γ* are crucial immune system components that prevent toxoplasmosis. It can also make *Toxoplasma* parasite easier to destroy and prevent the infection from reactivating in leukocytes like MQs [74]. Epitope is recognized by T-cells, B lymphocyte, and molecules in the immune system of host as a component of an antigen. Understanding the activities and structures of the intended antigen is possible through the analysis of epitopes, which is useful for determining the specificity and antigenicity of proteins. Additionally, epitope evaluation can be helpful in vaccine epitope studies [75] and may disclose disease and immunological pathways. The epitope prediction approach is primarily connected to various (not just one) features of the protein. Numerous noteworthy epitope index forecasts are accessible, such as mobility, hydrophilicity, antigenicity, and surface accessibility [20, 51, 53, 55]. Examining a single index alone does not provide enough or correct evidence on the target epitope. Consequently, an epitope is a peptide that may readily bind with an antibody and has the right indices to function as such. T-cell epitopes should therefore be chosen in addition to B-cell epitopes to offer protection against *T. gondii*. In light of the fact that the ROP29 protein sequences can trigger both cellular and humoral immune reactions, we attempted to identify immunodominant epitopes in this study. Because of the increase in precision and validity of this prediction, we used a several tools and servers in this research to anticipate T- and B-cell epitopes. ROP29 had positive epitopes and appropriate values, according to the outcomes of linear B-cell epitopes using ABCpred, BcePred, and BCPREDS online servers. For instance, BcePred's prediction accuracy ranges between 52.92% and 57.53% for models based on different features. Additionally, based on any physicochemical parameter (accessibility, turns, mobility, flexibility, hydrophilicity, polarity, and exposed surface), or a combination of features [35, 48], users of this server can anticipate B-cell epitopes. A further critical stage for in silico research is the discovery of conformational epitopes, which are essential for antibody-antigen binding. In this instance, the DiscoTope 2.0 web server discovered 13 discontinuous B-cell epitopes.

Using the IEDB online tool, we examined the IC_50_ values of ROP29-specific peptides that bind to MHC-II and I. Based on information from IEDB, it was shown that the T lymphocyte epitopes on ROP29 could bind strongly to MHC-II and I. Lower percentile scores (IC_50_ values) indicate higher level affinity, which is indicative of an improved T-cell epitope and vice versa. Furthermore, we used the CTLPred website to anticipate the CTL epitopes, and the 10 epitopes with the highest rankings were then chosen for the ROP29 protein. An essential component of vaccine design is CTL epitope forecasting, and CTLPred provides a realistic approach for doing so [57]. Toxoplasmosis is reliant on T-cell-mediated cellular immunity, since it is an obligate intracellular parasite [70]. To produce an effective vaccination against *T. gondii* infection, it is necessary to identify the precise kind of T-cell-mediated immune response that is involved [68, 70].

In the past, various studies have demonstrated that in vaccinated mice, ROPs can produce a durable immunity and stimulate T-cell and B-cell immunological responses with longer life time [24, 75]. The ROP29 protein was shown to be nonallergenic and being immunogenic, according to the results of the allergenicity and antigenicity assessments. Using several bioinformatic techniques, it is recommended to verify the immunogenicity of the projected sequences using a suitable animal model. Therefore, it is strongly advised to carry out additional research using in vivo techniques in the future to assess the effectiveness of protein as a potential vaccine candidate.

## 5. Conclusion

In this study, ROP29 protein's physicochemical properties, subcellular localization, potential T- and B-cell epitopes, secondary and tertiary structures transmembrane domain, and other features were recognized. Strong hydrophilicity, flexibility, surface accessibility, and antigenicity indexes have been demonstrated for ROP29. Additionally, the ROP29 protein had a number of excellent B- and T-cell epitopes, according to the results of epitope prediction using various bioinformatic servers, suggesting it would be a potential vaccine candidate against *T. gondii*. Our result showed that employing in silico methods to apply structural and functional predictions to the ROP29 protein can lower the likelihood that laboratory investigations will fail.

## 6. Suggestion

Future research is necessary to develop in vivo vaccines using ROP29 alone or in combination with other antigens.

## Figures and Tables

**Table 1:** Predicted acetylation sites of ROP29.

**Protein name**	**Acetylation**	**Peptide**	**Score**
ROP29	Yes	MSILG	0.508

**Table 2:** Predicted disulfide bonds by DINNA web.

Sequence inputSeq length 489 residues					
Cysteines in this sequence: 7					
Disulfide connectivity prediction					
Step 1: Running PSI-BLAST with input sequence; click here to see the output					
Step 2: Predicting secondary structure using PSIPRED; click here to see the output					
Step 3: Disulfide oxidation state prediction; click here to see the results					
Warning! The number of predicted half-cystines is lower than 2					
Step 4: Disulfide bond prediction using a trained neural network					
Disulfide bond scores					
Cysteine sequence position		Distance	Bond		Score
32–42		10	GGYGHCRDYLL-LQSESCWRGDL		0.01037
32–268		236	GGYGHCRDYLL-FPVDVCRISKL		0.01038
32–278		246	GGYGHCRDYLL-LFSGFCAGISY		0.01037
32–297		265	GGYGHCRDYLL-FPVFTCDLQQV		0.01038
32–408		376	GGYGHCRDYLL-LFLLWCGSFPY		0.01392
32–446		414	GGYGHCRDYLL-PNFHLCKDLPP		0.01757
42–268		226	LQSESCWRGDL-FPVDVCRISKL		0.01066
42–278		236	LQSESCWRGDL-LFSGFCAGISY		0.01037
42–297		255	LQSESCWRGDL-FPVFTCDLQQV		0.01037
42–408		366	LQSESCWRGDL-LFLLWCGSFPY		0.01037
42–446		404	LQSESCWRGDL-PNFHLCKDLPP		0.01044
268–278		10	FPVDVCRISKL-LFSGFCAGISY		0.01042
268–297		29	FPVDVCRISKL-FPVFTCDLQQV		0.01045
268–408		140	FPVDVCRISKL-LFLLWCGSFPY		0.01568
268–446		178	FPVDVCRISKL-PNFHLCKDLPP		0.95363
278–297		19	LFSGFCAGISY-FPVFTCDLQQV		0.01037
278–408		130	LFSGFCAGISY-LFLLWCGSFPY		0.01041
278–446		168	LFSGFCAGISY-PNFHLCKDLPP		0.02107
297–408		111	FPVFTCDLQQV-LFLLWCGSFPY		0.36613
297–446		149	FPVFTCDLQQV-PNFHLCKDLPP		0.01065
408–446		38	LFLLWCGSFPY-PNFHLCKDLPP		0.95448
Step 5: Weighted matching					
Predicted bonds					
32–42			GGYGHCRDYLL-LQSESCWRGDL		
268–446			FPVDVCRISKL-PNFHLCKDLPP		
297–408			FPVFTCDLQQV-LFLLWCGSFPY		
Predicted connectivity					
1–2, 3–7, 5–6					
Introduction	Instructions	References	About	Help!	Contacts
© Boston College					

**Table 3:** Prediction of epitopes on ROP29 protein using various criteria based on BcePred web service.

**Prediction parameter**	**Epitope sequence**
Flexibility	GDLTSGV; GVLTKKGTRARTTK; LQHLSSSQKK; ERFLREESSA; QVVKSGL; EAVPQEKTKTTASEERD; VTLGKTE; LQWNSDNRSRP
Hydrophilicity	TKKGTRARTTKRHD; HEEQTAPP; PVDTQGQLD; SSSQKKTA; REESSARNA; DPQTAEA; PQEKTKTTASEERDA; VSDEQADL; GKTEKPAPN; NSDNRSRPGD
Accessibility	TVGDRYKVRLTPEYG; RDYLLQS; RGVLTKKGTRARTTKRHDLV; GEHYWNQG; VYHEEQTAPP; DTQGQLD; NSVLKNRPPAEAQLSPREIAAKT; TLTKPAR; PQQLAAK; NVRSLQELQHLSSSQKKTALEDIQERFLREESSARNA; QHVTPET; HNDLKLEN; PYLDPQTAEAVPQEKTKTTASEERDAWA; PYVSDEQADLPT; TLGKTEKPAPNFH; LQWNSDNRSRPGDV
Turns	VAHNDLK; QWNSDNRSR
Exposed surface	DRYKVRL; KKGTRARTTKRHD; KNRPPAE; LSSSQKKTALED; VPQEKTKTTA; KTEKPAP; NSDNRSRP
Polarity	GDRYKVRLTPE; RGVLTKKGTRARTTKRHDLVA; GLVYHEEQTAP; KNRPPAE; PREIAAKTHFEAM; FGLHRVKVGG; QKKTALEDIQERFLREESSARN; PETALEHG; HNDLKLE; EAVPQEKTKTTASEERDAWA; TLGKTEKPAP; NSDNRSRP
Antigenic propensity	HCRDYLLQSESC; SGVRGVL; QGLVYHEEQ; LDINSVL; KGLQLTVESIF; IVFGLHRVKVG; VRSLQELQHLSSS; LEHGVLFPVDVCRISKLFSGFC; GISYVILPVL; LFPVFTCDLQQVVKSGLS; LYITRLLLQ; LKLENVLLS; TLLFLLWCGSFPYV; SDLLKLLVTLGK; PNFHLCKDLPP

**Table 4:** B-cell linear epitopes derived from full-length proteins using the ABCpred server.

**Rank**	**Sequence**	**Start position**	**Score**
1	GGGYGHCRDYLLQSESCWRG	26	0.91
2	VTAFLQWNSDNRSRPGDVLD	457	0.86
2	VESIFTGETFTLTKPARFLG	163	0.86
2	GDRYKVRLTPEYGPGGGYGH	12	0.86
3	WLHANGVAHNDLKLENVLLS	325	0.84
4	LFSGFCAGISYVILPVLALF	273	0.83
4	LQHLSSSQKKTALEDIQERF	218	0.83
5	VTSGEHYWNQGLVYHEEQTA	73	0.82
5	LFPVFTCDLQQVVKSGLSRA	291	0.82
6	TAPPVAASNSFMQSTTSLAT	91	0.81
6	SPREIAAKTHFEAMFAKGLQ	141	0.81
7	VLTKKGTRARTTKRHDLVAV	54	0.79
7	TSLATPADLNAPVDTQGQLD	106	0.79
8	PYLDPQTAEAVPQEKTKTTA	371	0.77
8	AKGLQLTVESIFTGETFTLT	156	0.77
8	PVDTQGQLDINSVLKNRPPA	117	0.77
9	GDLTSGVRGVLTKKGTRART	45	0.76

**Table 5:** Linear epitopes for B cells from full-length proteins predicted by the BCPREDS server.

**Position**	**Epitope**	**Score**
18	RLTPEYGPGGGYGHCRDYLL	1
88	EEQTAPPVAASNSFMQSTTS	0.999
374	DPQTAEAVPQEKTKTTASEE	0.991
109	ATPADLNAPVDTQGQLDINS	0.934
435	KTEKPAPNFHLCKDLPPAVG	0.914
167	FTGETFTLTKPARFLGWGST	0.898
188	IVFGLHRVKVGGASAPQQLA	0.814
404	FLLWCGSFPYVSDEQADLPT	0.758

**Table 6:** Using the IEDB^a^, IC_50_ values were determined for ROP29's affinity for MHC Class I.

**MHC-I allele** ^b^	**Start–stop**	**Peptide sequence** ^c^	**Percentile rank** ^d^
H2-Db	14–23	SAIQFTAPYL	0.14
	32–41	SSARNALQHV	0.475
	61–70	SAPQQLAAKI	0.48

H2-Dd	34–43	LTKPARFLGW	1.75
	61–70	SAPQQLAAKI	2.85
	23–32	YGPGGGYGHC	3.10

H2-Kb	14–23	SAIQFTAPYL	0.155
	1–10	IADFGFAVKL	0.475
	48–57	IVFGLHRVKV	1.85

H2-Kd	2–11	SYVILPVLAL	4.40
	28–37	GYGHCRDYLL	5.00
	39–48	RFLGWGSTAI	5.15

H2-Kk	10–19	HFEAMFAKGL	2.745
	16–25	YHEEQTAPPV	3.375
	11–20	FEAMFAKGLQ	3.875

H2-Ld	6–15	LPVLALFPVF	0.49
	5–14	ILPVLALFPV	1.60
	22–31	APPVAASNSF	2.80

^a^Database of immunological epitopes can be obtained at http://tools.iedb.org/mhci/.

^b^Mouse MHC Class I molecules are H2-Db, H2-Dd, H2-Kb, H2-Kd, H2-Kk, and H2-Ld alleles.

^c^Each time, 10 amino acids were employed for the analysis.

^d^High percentile rank, low-level binding; low percentile rank, high-level binding; and percentile rank, IC_50_ values.

**Table 7:** Using the IEDB^a^, IC_50_ values were determined for ROP29's affinity for MHC Class II.

**MHC-II allele** ^b^	**Start–stop**	**Peptide sequence** ^c^	**Percentile rank** ^d^
H2-IAb	15–29	VYHEEQTAPPVAASN	1.60
	29–43	NSFMQSTTSLATPAD	2.09
	16–30	YHEEQTAPPVAASNS	1.85

H2-IAd	29–43	NSFMQSTTSLATPAD	1.24
	27–41	ASNSFMQSTTSLATP	1.99
	30–44	SFMQSTTSLATPADL	2.05

H2-IEd	46–60	TAIVFGLHRVKVGGA	2.05
	45–59	STAIVFGLHRVKVGG	2.55
	48–62	IVFGLHRVKVGGASA	3.70

^a^The immune epitope dataset, located at http://tools.iedb.org/mhcii/.

^b^Three mouse MHC Class II molecules are known as H2-IAb, H2-IAd, and H2-IEd alleles.

^c^Each time, 15 amino acids were employed for analysis.

^d^High percentile rank = low level binding, low percentile rank = high level binding, and percentile rank = IC_50_ values.

**Table 8:** Predicted ROP29 epitopes by CTLPred^a^.

**Peptide rank**	**Start position** ^b^	**Sequence**	**Score (ANN/SVM)** ^c^	**Prediction**
1	397	WALATLLFL	0.72/1.2131132	Epitope
2	365	AIQFTAPYL	0.96/0.86668539	Epitope
3	350	VIADFGFAV	0.64/1.1689693	Epitope
4	281	ISYVILPVL	0.95/0.80020559	Epitope
5	312	KLYITRLLL	0.64/1.0911544	Epitope
6	309	RAAKLYITR	0.77/0.87313975	Epitope
7	164	ESIFTGETF	0.94/0.67417849	Epitope
8	184	GSTAIVFGL	0.96/0.56741988	Epitope
9	210	INVRSLQEL	0.87/0.64073564	Epitope
10	3	ILGKHTATV	0.97/0.5258532	Epitope

^a^CTLPred, a web service that is accessible online at http://crdd.osdd.net/raghava/ctlpred/.

^b^For the analysis, nine amino acids were employed.

^c^The default cutoff scores for support vector machines (SVMs) and artificial neural networks (ANNs) were set at 0.36 and 0.51, respectively.

## Data Availability

The data used to support the findings of this study are available from the corresponding author upon reasonable request.
